# Ribonucleotide reductase subunit M2 promotes proliferation and epithelial–mesenchymal transition via the JAK2/STAT3 signaling pathway in retinoblastoma

**DOI:** 10.1080/21655979.2021.2001241

**Published:** 2021-12-11

**Authors:** Min Yang, Panpan Yao, Xuqiang Lang, Xue Li, Dawei Zhang

**Affiliations:** a Department of Ophthalmology, Beijing Luhe Hospital, Capital Medical University, Beijing, China; b Department of Ophthalmology Medicine, Wusong Branch, Zhongshan Hospital, Fudan University, Shanghai, China

**Keywords:** RRM2, JAK2/STAT3, EMT, retinoblastoma

## Abstract

Retinoblastoma (RB) is an intraocular malignant tumor that often occurs in children. Along with the improvement of treatment strategies, the cure rate of RB has increased significantly. However, the treatment of advanced and recurrent RB remains as a critical challenge. Therefore, studying the molecular mechanisms underlying the progression of RB is essential for the development of novel and effective therapeutic strategies. Through the analysis of a previously published microarray study, we found that ribonucleotide reductase subunit M2 (RRM2) was highly expressed in RB tissues as compared to normal tissues. The purpose of this study is to clarify the role and mechanism of RRM2 in regulating the progression of RB. We first demonstrated that RRM2 expression level in RB tissues and cell lines was significantly higher when compared to that in normal retinal tissue and cell lines, and high RRM2 expression level was associated with a poorer overall survival of patients. In RB cells, RRM2 overexpression promoted cell proliferation, migration, invasion and epithelial–mesenchymal transformation (EMT), while RRM2 silencing suppressed these biological features. Silencing RRM2 reduced the activation of Janus kinase 2 (JAK2)/signal transducer and activator of transcription 3 (STAT3) signaling pathway, and the presence of JAK2/STAT3 signaling pathway inhibitor INCB attenuated the effect of RRM2 overexpression. Collectively, our data indicate that RRM2 promotes the progression of RB by activating JAK2/STAT3 signaling pathway. Targeting RRM2/JAK2/STAT3 axis lays a theoretical foundation for the formulation of novel RB therapy.

## Introduction

Retinoblastoma (RB) is a type of malignant tumor that often occurs in the eyes of children under 3 years old. It originates from the embryonic neural retina and occurs in any part of the retinal nucleogranule. RB seriously affects children’s vision and threatens their lives [1,2]. Optic nerve invasion and distant metastasis of RB cells are crucial factors leading to poor prognosis of RB patients, especially in developing countries [3]. At present, chemotherapy and local treatment are the major therapeutic strategies in RB [4]. With the development of novel therapeutic approaches, the treatment outcome of RB has been significantly improved. However, the treatment efficacy of refractory and recurrent tumors is limited.

Ribonucleotide reductase (RR) is key enzyme mediating the synthesis of deoxyribonucleotides for DNA precursors, which are indispensable in cell cycle progression, DNA replication and repair. It consists of two subunits, Ribonucleotide reductase M1 subunit (RRM1) and RRM2. RRM2 serves as the catalytic subunit of RR, and its relative abundance affects the DNA replication and cell proliferation [5,6]. A large amount of evidence shows that RRM2 functions as an oncogenic factor to promote the progression of various tumors [7,8], regulate tumor cell differentiation and metastasis [9], and modulate chemotherapy resistance [10]. RRM2 expression level seems to associate with tumor grade, and RRM2 has been proposed as a biomarker for diagnosis and prognosis [11–13]. However, whether RRM2 is implicated in RB progression is unclear.

Janus kinase 2 (JAK2)/signal transducer and activator of transcription 3 (STAT3) signaling pathway is responsible for transmitting extracellular signals through cell surface receptors, thereby regulating downstream target genes in a myriad of biological processes including cell proliferation, cell death, and immune responses [14]. The constitutive activation of this signaling pathway is frequently found in a variety of cancers to facilitate tumor progression and drug resistance development, such as breast cancer [15,16], gastric cancer [17], and colon cancer [18]. Epithelial–mesenchymal transformation (EMT) is a program in which epithelial cells transform into mesenchymal phenotype with enhanced cell migration and invasion capabilities [19]. It is accompanied by the expression of a series of transcription factors related to EMT, including SNAIL, ZEB and TWIST [19]. The activation of JAK2/STAT3 signaling pathway has been reported to induce EMT and metastasis in breast cancer [20]. However, little is known about the role of JAK2/STAT3 signaling pathway in RB.

This study aims to investigate the functional role and underlying mechanism of RRM2 in the development of RB. We compared RRM2 expression in RB tissue and normal retinal tissue, and further analyzed the correlation between RRM2 expression level and the overall survival of RB patients. The results illustrated that RRM2 was highly expressed in RB tissues and cell lines, and high RRM2 expression level was associated with a poorer overall survival of patients. We then investigated the potential oncogenic role of RRM2 in RB cell proliferation, migration, invasion as well as EMT by knocking down or overexpressing RRM2. We further explored the downstream signaling pathway of RRM2 and revealed the crucial role of JAK2/STAT3 signaling pathway in RRM2-mediated oncogenic effect. Collectively, our study provides novel insights into the role of RRM2 in RB progression and identifies JAK2/STAT3 axis as a downstream signaling pathway in RRM2-mediated oncogenic effect.

## Materials and methods

### Tissue samples

A total of 20 cases of normal retinal tissues and 46 cases of retinoblastoma tissues were collected in the Department of Ophthalmology, Beijing Luhe Hospital, Capital Medical University. For normal retinal tissues, postmortem human retina tissues from donors were obtained in our hospital after obtaining written informed consent of the next-of-kin. The human retina tissues were obtained and snap-frozen in liquid nitrogen within 4 h postmortem. All RB patients who provided the retinoblastoma tissues signed the informed consent. All the experimental procedures were approved by the Medical Ethics Committee of Beijing Luhe Hospital, Capital Medical University.

### Cell culture and transfection

DMEM basic medium (Thermo Fisher Scientific, Gaithersburg, MD, USA) was used to culture human RB cell lines Y79 and WERI-Rb1, and DMEM/F12 basic medium was used to culture normal retinal epithelial cell-line ARPE-19. All cell culture media were supplemented with 10% fetal bovine serum (FBS; Thermo Fisher Scientific/Gibco) and 100 U/mL penicillin, and 100 µg/mL streptomycin.

For transient transfection, a total number of 5 × 10^6^ cells of Y79 and WERI-Rb1 in 200 uL Opti-MEM medium were electroporated with 10 µg pcDNA3.1-RRM2 plasmids, or 100 nM si-RRM2#1/si-RRM2#2 or the corresponding controls employing Electroporator II (Invitrogen, Carlsbad, CA, USA) at a condition of 200 V, 1000 mF. After electroporation, cells were cultured in complete medium for additional 44 hours before subsequent experiments. siRNA and pcDNA3.1-RRM2 overexpression plasmids were purchased from RiboBio Co., Ltd. (Guangzhou, China).

For stable knockdown, cells were infected with recombinant lentiviruses carrying short hairpin RNA (shRNA) targeting human RRM2 (sh-RRM2) or negative control (sh-NC) construct according to the manufacturer’s protocol. Recombinant lentiviruses and negative control were produced and purchased from Genechem Co., Ltd. (Shanghai, China). For inhibiting the activity of JAK2/STAT3 signaling pathway, JAK2 inhibitor INCB (Ruxolitinib INCB018424, Selleckchem, Seoul, Republic of Korea) was added into the medium at the concentration of 5 µM.

### Quantitative PCR analysis

The total RNA in tissues and cells were extracted by TRIZOL reagent (Thermo Fisher Scientific), and the concentration and purity of RNA were measured by Nanodrop spectrometer. SuperScript First Strand cDNA System (Invitrogen) was used for cDNA synthesis from 5 μg total RNA. Subsequently, SYBR® Premix DimerEraser™ reagent (Takara, Dalian, China) was used to perform the quantitative PCR assay on the 7500 Real-Time PCR System (Applied Biosystems/Life Technologies, Carlsbad, CA, USA). The results were analyzed using 2^–∆∆Ct^ method, with GAPDH as the internal reference gene [21]. The primers were synthesized by Shanghai Sangon Biotechnology Co., Ltd. (Shanghai, China) as follows:

RRM2:

Forward: 5ʹ-CACGGAGCCGAAAACTAAAGC-3ʹ

Reverse:5ʹ-TCTGCCTTCTTATACATCTGCCA-3ʹ

GAPDH:

Forward: 5ʹ-AGAGATGCCATTCTGGCC-3ʹ

Reverse: 5ʹ-GTGGAGTAGAAATGCTGG-3ʹ

### Western blot

Cells in logarithmic growth phase were washed twice with phosphate-buffered saline (PBS), and were lyzed by RIPA Lysis Buffer on ice for 15 minutes. Cell debris was removed by centrifugation, and the concentration of protein was measured by a BCA kit (Thermo Fisher Scientific). 20 ug of proteins were separated by SDS-PAGE and transferred to polyvinylidene fluoride (PVDF) membrane. The membrane was blocked with 5% skimmed milk for 1 hour, and was further incubated with the primary antibodies: anti-RRM2 (CST, #65,939, 1:1000), anti-snail1 (CST, #3879, 1:1000), anti- E-cadherin (CST, #14,472, 1:1000), anti-N-cadherin (CST, #13,116, 1:1000), anti-JAK2 (CST, #3230, 1:1000), anti-p-JAK2 (CST, #66,245, 1:1000), anti-STAT3 (CST, #9139, 1:1000), anti-p-STAT3 (CST, #9145, 1:1000) and anti-GAPDH (CST, #5174, 1:1000) at 4°C overnight. The next day, the membrane was washed with TBST buffer for 3 times and was incubated in TBST buffer containing HRP-conjugated secondary antibody (CST, #7074, 1:2000; CST, #7076, 1:2000) for 1 hour. The membrane was washed with TBST buffer for 4 times and protein bands were developed using enhanced chemiluminescence reagent (Thermo Fisher Scientific) in a dark room. The densitometry of protein bands were analyzed as previously described [22].

### CCK8 cell proliferation assay

Y79 and WERI-Rb1 cells with indicated treatments were seeded into a 96-well plate at a density of 2000 cells/well and cultured in a cell incubator for 0 h, 24 h, 48 h, 72 h. 10 μL CCK8 reaction solution (Solarbio, Beijing, China) was added to each well at indicated time point. The cell culture was incubated in cell incubator for 4 h, and the absorbance at 450 nm was measured by a microplate reader (Bio-Tek Instrument, Winooski) [23].

### Colony formation experiment

Cells were trypsinized and resuspended in the culture medium. After cell counting, cells were seeded into a 6-well plate (1000 cells/well) and cultured for 14 days. During this period, the culture medium was changed every 3 days. After 14 days, cells were fixed with 4% paraformaldehyde for 15 minutes at 4°C and stained with 0.5% crystal violet dye (Sigma-Aldrich, St. Louis, Missouri, USA) for 20 minutes. The stained colonies were photographed and counted under a light microscope [24].

### Cell invasion and migration assay

The Transwell chamber (Corning, NY, USA) was used to evaluate cell migration and invasion capabilities. Transwells not coated with Matrigel matrix (BD Biosciences, Bedford, MA) were used for migration assay, and Transwells coated with Matrigel matrix are used for invasion assay. Cells in logarithmic growth phase were trypsinized and resuspended with serum-free medium. 100 µL of cell suspension containing 5 × 10^5^ cells were added into the upper chamber of Transwells and 500 μL culture medium containing 10% FBS was added to the lower chamber. After 24 hours, cells on the Transwell membrane were fixed with 4% paraformaldehyde for 30 minutes, and stained with 0.5% crystal violet dye. Migrating and invading cells were counted under the light microscope [22].

### In vivo *tumor formation assay*

A total of 1 × 10^7^ Y79 cells with stable RRM2 knockdown (sh-RRM2) and the control cells (sh-NC) were inoculated subcutaneously in BALB/c nude mice (6 weeks old, n = 6 in each group). The size of the subcutaneous tumor was measured at the indicated time point for 4 weeks. At day 28, the mice were sacrificed and the tumors were removed and weighed. Protein sample of the tumor tissues were extracted and detected by Western blot. All experimental procedures were approved by the Laboratory Animal Ethics Committee at the Beijing Luhe Hospital, Capital Medical University.

### Statistical analysis

SPSS 13 statistical software (SPSS Inc., Chicago, IL, USA) was used for statistical analysis, and GraphPad Prism 6.0 (https://www.graphpad.com/) was used for preparing the figures. All data are presented as mean ± standard deviation (SD). Unpaired Student’s t-test was used to compare the difference between the treatment group and control group, and two-way analysis of variance (ANOVA) was used to compare the difference in tumor volume between the treatment group and control group. A value of *P* < 0.05 was considered to be statistically significant.

## Results

This study aims to elucidate the function and underlying mechanism of RRM2 in the progression of RB. We first analyzed the previously published microarray dataset (GSE97508 in Gene Expression Omnibus database), and found that RRM2 was upregulated in RB tissues. The upregulation of RRM2 was further confirmed in RB tissues from patients and a high expression level of RRM2 was associated with a poor prognosis for patients. The oncogenic role of RRM2 was validated in RB cell lines by loss-of-function and gain-of-function experiment *in vitro* and *in vivo*. Lastly, we revealed that JAK2/STAT3 axis functions as a downstream signaling pathway in RRM2-mediated oncogenic effect.

### RRM2 is highly expressed in RB tissues and cell lines

**Figure 1.**
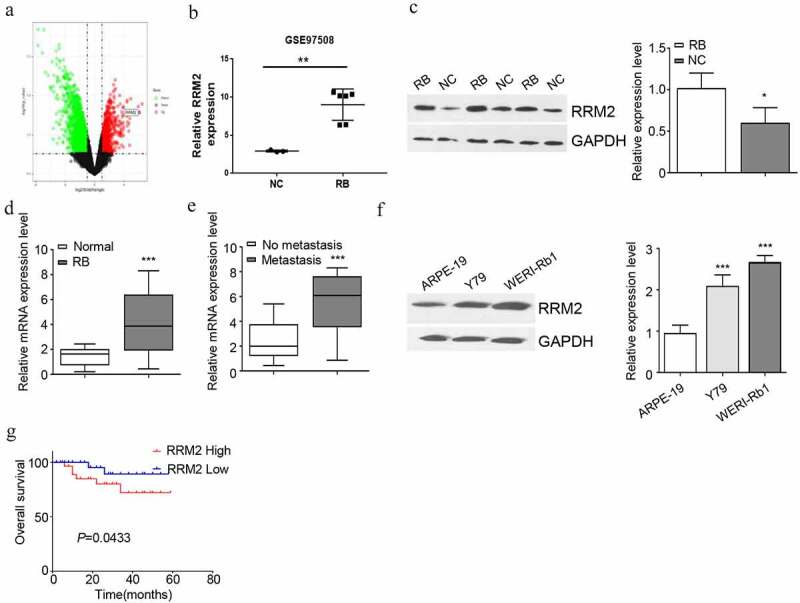
RRM2 is highly expressed in RB cancer tissues and cells. (a) Volcano plot of differential gene expression from GSE97508 data in Gene Expression Omnibus database. (b) Expression level of RRM2 using data from GSE97508 data.(c) The difference of RRM2 protein expression in retinoblastoma (RB) and normal retina tissues was detected by Western blot. (d) The difference of RRM2 mRNA expression in RB samples (n = 46) and normal retina tissue (n = 20) was detected by RT-qPCR. (e) The difference of RRM2 mRNA expression between metastatic RB and non-metastatic RB was detected by RT-qPCR. (f) The protein expression of RRM2 in human RB cell lines Y79, WERI-Rb1 and normal retinal cell line ARPE-19 was detected by Western blot. (g) The median expression value of RRM2 in 46 cases of RB patients was used as the cutoff value to divide into high and low expression group. The relationship between RRM2 expression level and the overall survival rate of RB patients was analyzed by Kaplan Meier Curve and log-rank test. Three independent assays of RT-qPCR were performed with three technical replicates. *, P < 0.05, **, P < 0.01, and ***, P < 0.001

To explore the function of RRM2 in RB, we analyzed GSE97508 dataset from Gene Expression Omnibus database. TRRM2he volcano plot revealed that RRM2 was a highly significantly up-regulated gene in RB tissues (Figures 1A, 1B). To further confirm the regulation of RRM2 in RB, we collected 20 cases of normal retinal tissues (NC) and 46 cases of RB tissues. qRT-PCR and Western blot analysis showed that compared with normal retinal tissues, RRM2 expression level in RB tissues was significantly increased (Figures 1C, 1D). The 46 cases of RB tissues were divided to metastasis group (n = 16) and no metastasis group (n = 30). qRT-PCR analysis showed the dramatic upregulation of RRM2 in metastatic group as compared to the non-metastatic ones (Figure 1e). Using RB cell lines (Y79 and WERI-Rb1) and normal retinal epithelial cell line (ARPE-19), we further showed that a significantly higher expression of RRM2 in RB cell lines (figure 1f). To investigate whether RRM2 expression level is associated with patient survival, 46 RB patients were divided into high expression (n = 23) and low expression group (n = 23) based on the median expression value of RRM2. We found that a high expression level of RRM2 was associated with a poor prognosis in RB patients (Figure 1g). We also evaluated the relationship between RRM2 expression and clinical parameters. High RRM2 expression is closely related to tumor size and TNM stage, but not to the patient’s age and gender (Table 1). Together, the above results indicate that elevated RRM2 expression may contribute to the progression of RB.

**Table 1.** The relationship between RRM2 and clinical parameters

Parameters	RRM2 expression		P
	High (n = 23)	Low (n = 23)	
Sex			0.555
male	13 (56.52)	11 (47.83)	
female	10 (43.48)	12 (52.17)	
Age			0.326
<5 years	15 (65.22)	18 (78.26)	
≥5 years	8 (34.78)	5 (21.74)	
TNM stage			0.074
I – II	10 (43.48)	16 (69.57)	
III – IV	13 (56.52)	7 (30.43)	
Tumor size (mm)			0.077
≤15	9 (39.13)	15 (65.22)	
>15	14 (60.87)	8 (34.78)

### RRM2 silencing suppresses the proliferation, migration, invasion and EMT of RB cells

**Figure 2.**
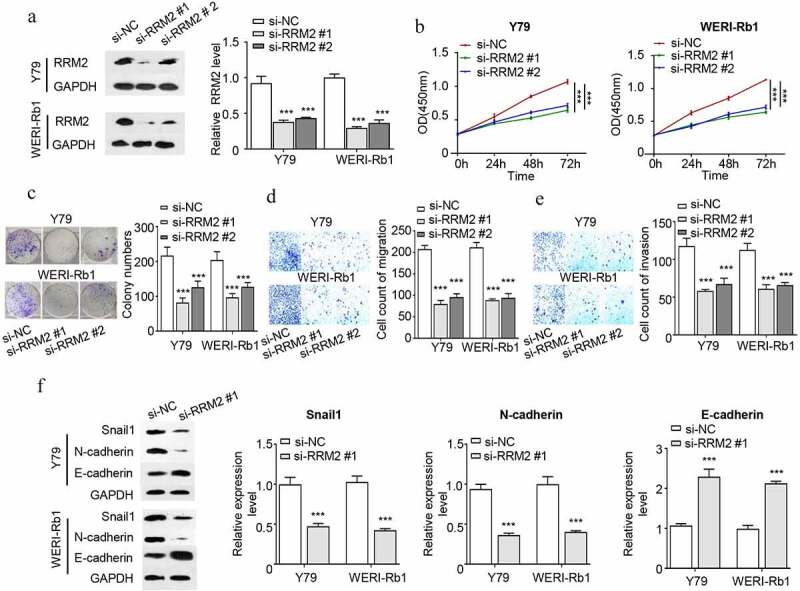
Interfering with the expression of RRM2 decreased the proliferation, EMT migration and invasion of Rb cells. (a) The silencing efficiency of RRM2 in RB cells Y79 and WERI-Rb1 was assessed by Western blot. B The light absorption value of Y79 and WERI-Rb1 cells at 450 nm wavelength was detected by CCK8 assay. (c) The colony forming ability of Y79 and WERI-Rb1 cells with different treatments was evaluated by colony forming assay. (d) and (e) The migration (d) and invasion (e) ability of Y79 and WERI-Rb1 cells with indicated treatment was examined by Transwell assay. (f) The expression levels of EMT-related proteins (snail1, E-cadherin and N-cadherin) in Y79 and WERI-Rb1 cells were detected by Western blot. Three independent assays were performed. *, P < 0.05, **, P < 0.01, and ***, P < 0.001

In order to validate the requirement of RRM2 in the malignant phenotype RB cells, Y79 and WERI-Rb1 cells were transfected with si-RRM2 #1 or si-RRM2 #2 or the control si-NC. Transfection of si-RRM2 #1 and si-RRM2#2 could effectively reduce RRM2 expression (Figure 2a). CCK8 proliferation assay demonstrated that the knockdown of RRM2 significantly impaired cell proliferation of Y79 and WERI-Rb1 cells (Figure 2b). Colony formation experiment showed that knocking down RRM2 dramatically suppressed the clonogenic ability of RB cells (Figure 2c). Silencing RRM2 also significantly attenuated the migration and invasion ability of RB cells (Figure 2d and e). Since epithelial–mesenchymal transition (EMT) is an important contributor to cancer cell migration, we next performed Western blot to assess the expression of EMT markers upon RRM2 silencing. Knocking down RRM2 significantly reduced snail1 and N-cadherin expression (mesenchymal markers), while promoted E-cadherin expression (epithelial marker) (figure 2f). Together, the above results indicate that RRM2 is indispensable for the malignant phenotype of RB cells.

### Overexpression of RRM2 promotes the proliferation, migration, invasion and EMT of RB cells

**Figure 3.**
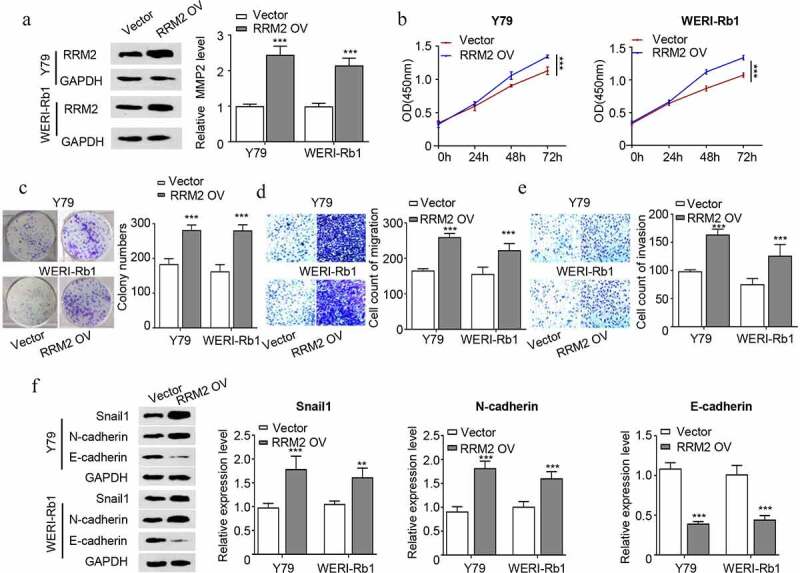
Overexpression of RRM2 enhances the proliferation, migration and invasion and EMT of RB cells. (a) The overexpression efficiency of RRM2 in Y79 and WERI-Rb1 cells was detected by Western blot. (b) The changes in the proliferation ability of Y79 and WERI-Rb1 cells after overexpressing RRM2 was analyzed by CCK8. (c) The clonogenic ability of Y79 and WERI-Rb1 cells after overexpressing RRM2 was detected by the colony forming assay. (d) and (e) The changes in migration (d) and invasion (e) ability of Y79 and WERI-Rb1 cells after RRM2 overexpression were examined by Transwell assay. (f) The changes in the protein levels of EMT-related proteins (snail1, E-cadherin and N-cadherin) after overexpression of RRM2 were detected by Western blot. Three independent assays were performed. *, P < 0.05, **, P < 0.01, and ***, P < 0.001

We next performed gain-of-function analysis by transfecting RB cells with pcDNA3.1-RRM2 expression plasmid (RRM2 OV) and control plasmid pcDNA3.1 (Vector). pcDNA3.1-RRM2 transfection increased RRM2 by approximately 2 folds in both Y79 and WERI-Rb1 cells (Figure 3a). Functional assays demonstrated that RRM2 overexpression promoted cell proliferation (Figure 3b), colony formation ability (Figure 3c), cell migration (Figure 3d) and cell invasion ability (Figure 3e) of Y79 and WERI-Rb1 cells. We also detected EMT markers by Western blot and the results showed that overexpression of RRM2 enhanced snail1 and N-cadherin expression, but suppressed E-cadherin expression (figure 3f). The above results overall suggest that overexpression of RRM2 is sufficient to strengthen the malignant phenotype of RB cells.

### RRM2 promotes the proliferation, migration and invasion of RB cells via the JAK2/STAT3 pathway

**Figure 4.**
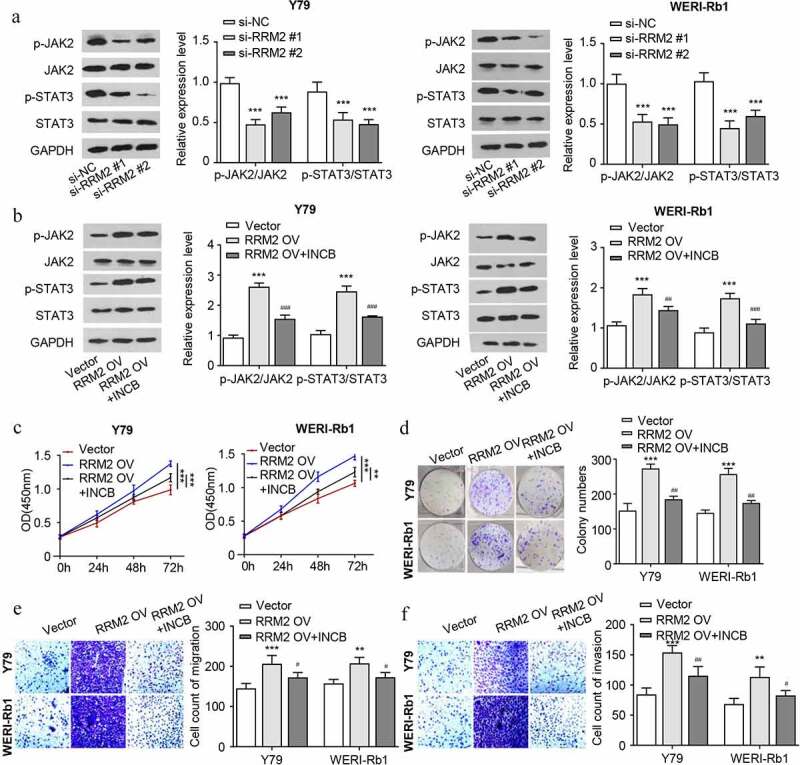
RRM2 regulates proliferation, invasion and EMT via the JAK2/STAT3 pathway in retinoblastoma cells. (a) The protein levels of JAK2/STAT3 pathway molecules (JAK2, p-JAK2, STAT3 and p-STAT3) in Y79 and WERI-Rb1 cells after RRM2 silencing by Western blot. B. The protein levels of JAK2/STAT3 pathway molecules were detected after RRM2 overexpression. JAK2 inhibitor INCB (5 µM) was used to inhibit the JAK2/STAT3 signaling pathway. C. The CCK8 proliferation assay was used to detect the proliferation ability after different treatments. D. The clonogenic ability of cells were evaluated by colony forming assay. E and F. The migration (d) and invasion (e) ability of Y79 and WERI-Rb1 cells were examined by Transwell assay. Three independent assays were performed. *, P < 0.05, **, P < 0.01, and ***, P < 0.001

In order to explore the potential mechanism of RRM2-mdeiated cell phenotype in RB, we detected the protein levels of JAK2/STAT3 pathway molecules in RB cells. The results showed that the ratio of p-JAK2/JAK2 and p-STAT3/STAT3 decreased after knocking down RRM2 (Figure 4a), which indicates that RRM2 is required to maintain the phosphorylation of JAK2 and STAT3. In contrast, the ratio of p-JAK2/JAK2 and p-STAT3/STAT3 increased after RRM2 overexpression, and the treatment with JAK2 inhibitor INCB attenuated the phosphorylation JAK2 and STAT3 induced by RRM2 (Figure 4b). To investigate the functional involvement of JAK2/STAT3 pathway in the oncogenic effect of RRM2, we further performed functional assays upon RRM2 overexpression with or without INCB treatment. The results showed that the oncogenic effects of RRM2 overexpression such as cell proliferation, colony formation, migration and invasion were significantly attenuated by JAK2 inhibitor INCB (Figure 4c, D, E and F). Collectively, our data suggest that the JAK2/STAT3 signaling pathway mediates the oncogenic roles of RRM2 in RB cells.

### Knocking down RRM2 suppresses the tumorigenesis of RB cells in nude mice

**Figure 5.**
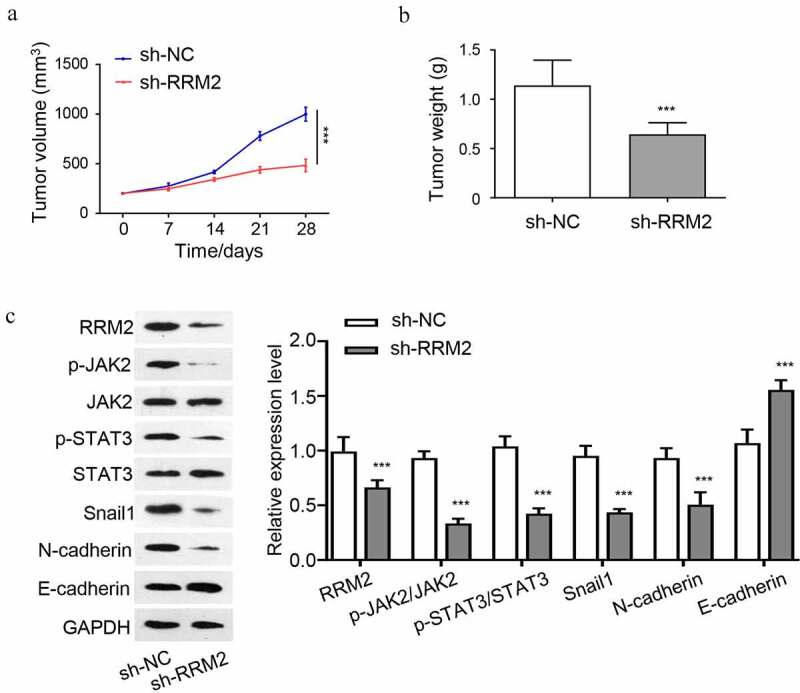
**Knockdown of RRM2 inhibits the tumorigenesis of RB cells in nude mice**. 1 × 10^7^ Y79 cells with stable RRM2 knockdown (sh-RRM2) or the control cells (sh-NC) were inoculated subcutaneously in nude mice. (a) The size of xenograft tumors was measured every 7 days. (b) The subcutaneous tumor weight of different groups (sh-NC and sh-RRM2) was measured at the end of the experiment. C. The protein levels of JAK2, p-jak2, STAT3, p-STAT3, RRM2, snail1, N-cadherin and E-cadherin in tumor samples of sh-NC and sh-RRM2 groups were detected by Western blot. *, P < 0.05, **, P < 0.01, and ***, P < 0.001

To further validate the oncogenic role of RRM2 *in vivo*, we performed tumorigenesis xenograft experiment by inoculating Y79 cells silenced with RRM2 shRNA (sh-RRM2) or the control cells (sh-NC) into nude mice. The knockdown of RRM2 significantly suppressed the tumorigenesis of Y79 cells in nude mice, as revealed by the suppressed tumor volume increase (Figure 5a) and the reduced tumor weight (Figure 5b). We also analyzed the JAK2/STAT3 pathway and EMT markers in the tumor samples by Western blot. The results showed that protein levels of RRM2, snail1 and N-cadherin were reduced in sh-RRM2 group, while E-cadherin was increased (Figure 5c). The phosphorylation level of JAK2 and STAT3 were also reduced in sh-RRM2 group. Together, the above results indicate that the oncogenic role of RRM2 in tumorigenesis and EMT is mediated by JAK2/STAT3 signaling pathway.

## Discussion

In this study, our data illustrated that RRM2 expression in RB tissues is significantly higher than that of normal retinal tissues, which seems to correlate with a poor survival of RB patients. Our data support that RRM2 exerts a oncogenic role in RB cells by activating ATK2/STAT3 signaling pathway through maintaining high level of phosphorylation. The oncogenic role of RRM2 could significantly attenuated by JAK2 inhibitor.

RR is highly expressed in tumors to support accelerated cell proliferation and metastasis. In recent years, various RR inhibitors have been developed for tumor treatment [25–27]. As the catalytic subunit of RR, RRM2 is the rate-limiting enzyme for DNA synthesis and repair, and its expression level is associated with the sensitivity of cancer cells to chemotherapy drugs and endocrine therapy drugs [10,25,28]. Previous studies indicated that in neuroblastoma, a highly malignant brain tumor, patients with high RRM2 expression are associated with a worse prognosis [29]. A previous study also suggests that RRM2 upregulation contributes to RB cell cycle progression [30]. Consistently, our study validated the oncogenic role of RRM2 in RB tissues, cell lines and xenograft tumorigenesis mouse model.

In terms of the regulation of RRM2 expression, previous studies indicate that LncRNA/miRNA axis regulates RRM2 expression. For example, LINC00667/miR-143-3p axis controls Non-Small Cell Lung Cancer progression through targeting RRM2 [31]. In addition, SNHG16/miR-30a/RRM2 axis also accelerates breast cancer cell proliferation and promote cell invasion [32], while miR-30a regulates liver cell proliferation and apoptosis through the suppressor of cytokine signaling 1 (SOCS-1)/JAK/STAT signaling pathway in rats with sepsis [33]. The mechanism underlying RRM2 upregulation in RB remains to be determined.

The JAK2/STAT3 signaling pathway mediates the function of solute carrier family 6 member 14 (SLC6A14) to promote the proliferation and metastasis of colorectal cancer cells [34]. Interestingly, a previous study showed that when JAK2/STAT3 signaling pathway is activated, cancer cells tend to escape the immune surveillance and metastasize to distant organs through blood circulation, resulting in the formation of metastatic lesions [35]. In ovarian cancer, the activation of JAK2/STAT3 signaling is also correlated with enhanced EMT and a series of malignant phenotypes [36]. The above findings and our data both support that JAK2/STAT3 signaling activation promotes the progression and metastasis of cancer. As an important signal transducer, STAT3 is phosphorylated upon external receptor activation and translocates into the nucleus to modulate target gene expression [37,38]. A previous study has found that RRM2 promotes breast cancer progression via activating phosphatidylinositol 3-kinase (PI3K)/AKT signaling pathway [39]. Both the activation of PI3K/AKT and JAK2 can phosphorylate STAT3 [40–43]. Our data showed that RRM2 overexpression promotes the phosphorylation of JAK2 and STAT3, which seems to be required for its oncogenic role in RB cell. However, it remains to be elucidated how RRM2 modulate the activation of JAK2 and STAT3.

RRM2 inhibitor Osalmid has been shown to enhance the radiosensitivity of esophageal cancer [44]. The application of JAK/STAT3 inhibitors also greatly enhances the sensitivity of colorectal cancer cells to chemotherapy [45]. JAK2 inhibitor XL019 promotes the apoptosis of vincristine-treated resistant human oral squamous carcinoma cells [46]. However, the effects of RRM2 inhibitors and JAK2/STAT3 inhibitors in RB therapy have not been reported. In our study, we showed that the treatment with JAK2 inhibitor INCB attenuates the malignant phenotype of RN cells upon RRM2 overexpression. However, whether JAK2/STAT3 inhibitor could synergize with current RB therapeutics will need to be evaluated in animal model and clinical trials.

## Conclusions

In summary, our study elucidates the oncogenic role of RRM2 in RB progression. The oncogenic role of RRM2 was validated in RB cell lines by loss-of-function and gain-of-function experiment. RRM2 supports the malignant phenotype of RB cells by maintaining the activation of JAK2/STAT3 signaling pathway. Future study is required to investigate whether RRM2 inhibitor could synergize with JAK2/STAT3 inhibitors to enhance the anticancer treatment of RB.

## Supplementary Material

Supplemental Material

## Highlights

- RRM2 functions as an oncogenic factor in RB.
- RB patients with high RRM2 expression are associated with poor prognosis.
- RRM2 promotes proliferation and metastasis of RB cells through JAK2/STAT3 signaling pathway.
