# Supplementary material for: Ribonucleotide reductase subunit M2 promotes proliferation and epithelial–mesenchymal transition via the JAK2/STAT3 signaling pathway in retinoblastoma
Source: Bioengineered. 2021 Dec 11;12(2):12800–11. doi: 10.1080/21655979.2021.2001241 (PMC8809947; doi:10.1080/21655979.2021.2001241)
Supplement: Supplemental Material [file KBIE_A_2001241_SM5412.pdf]

Figure 1C

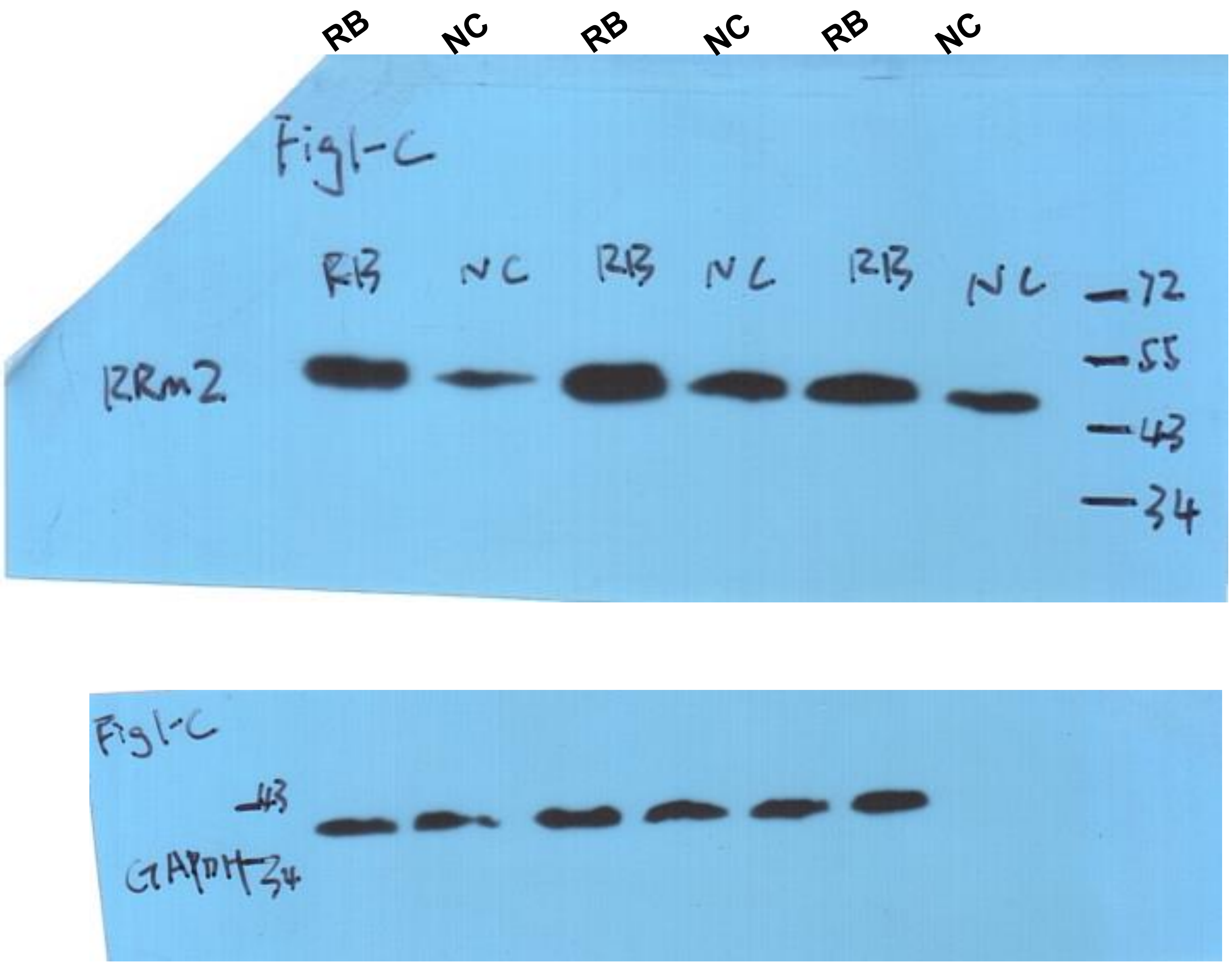

Figure 1F

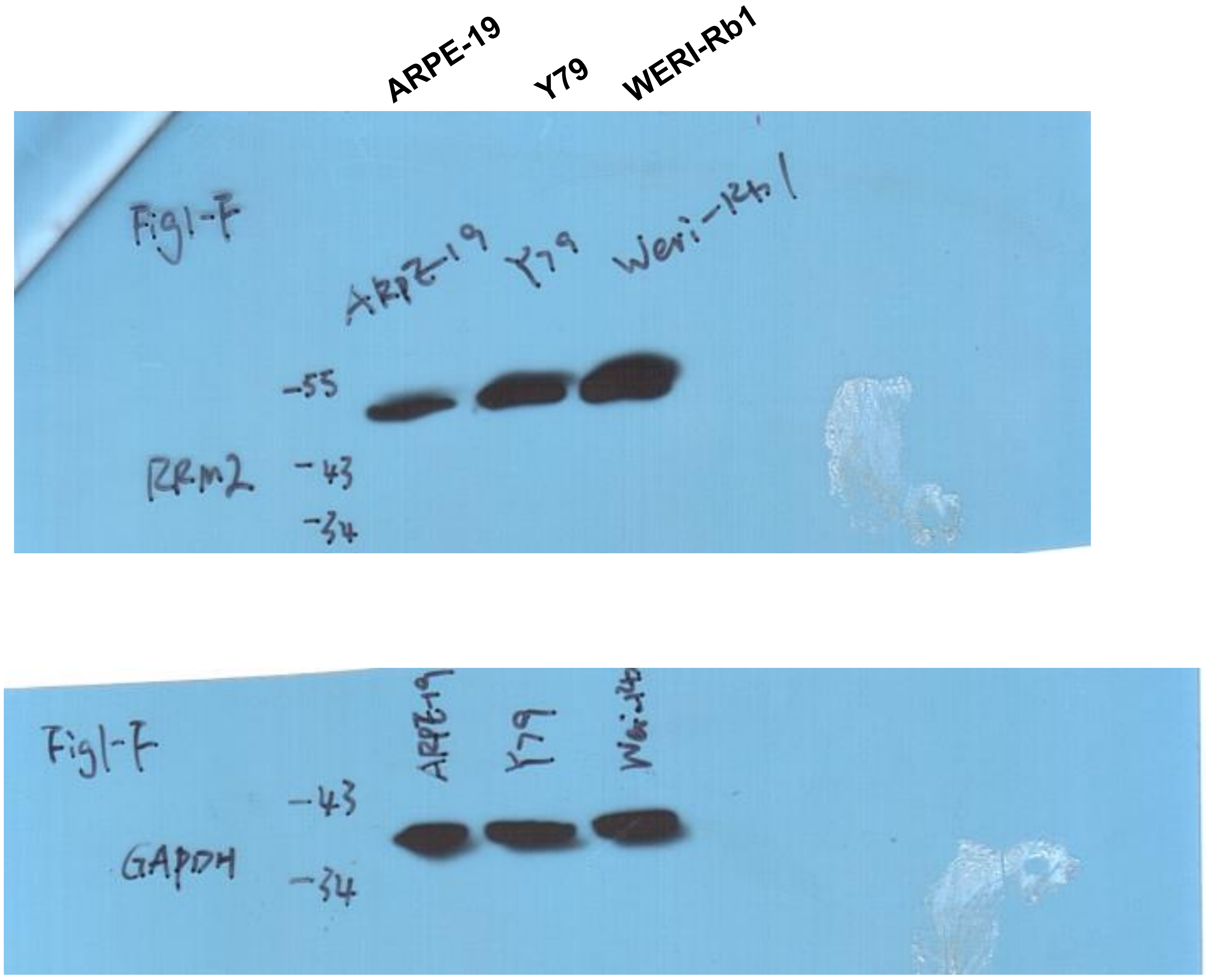

Figure 2A

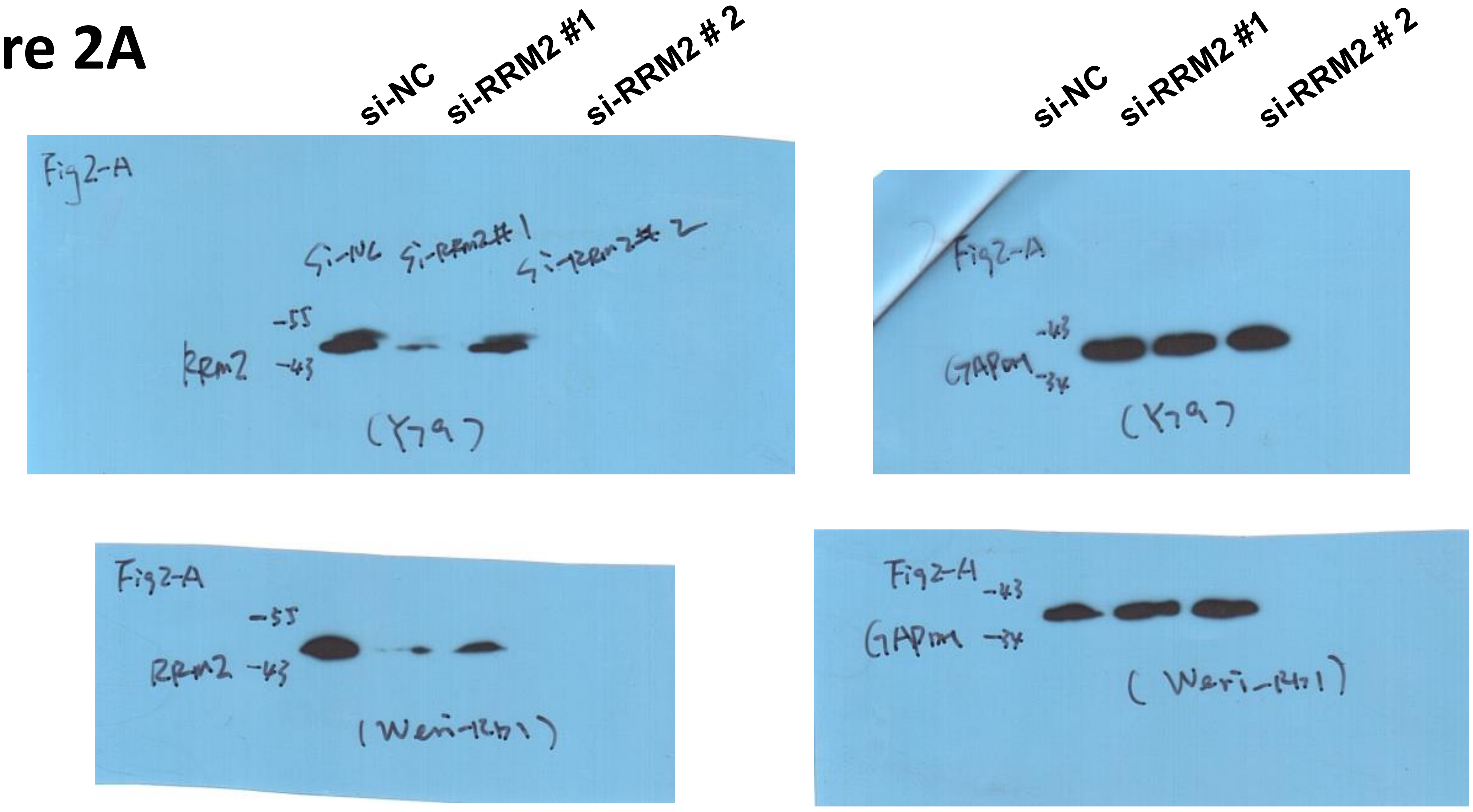

Figure 2F

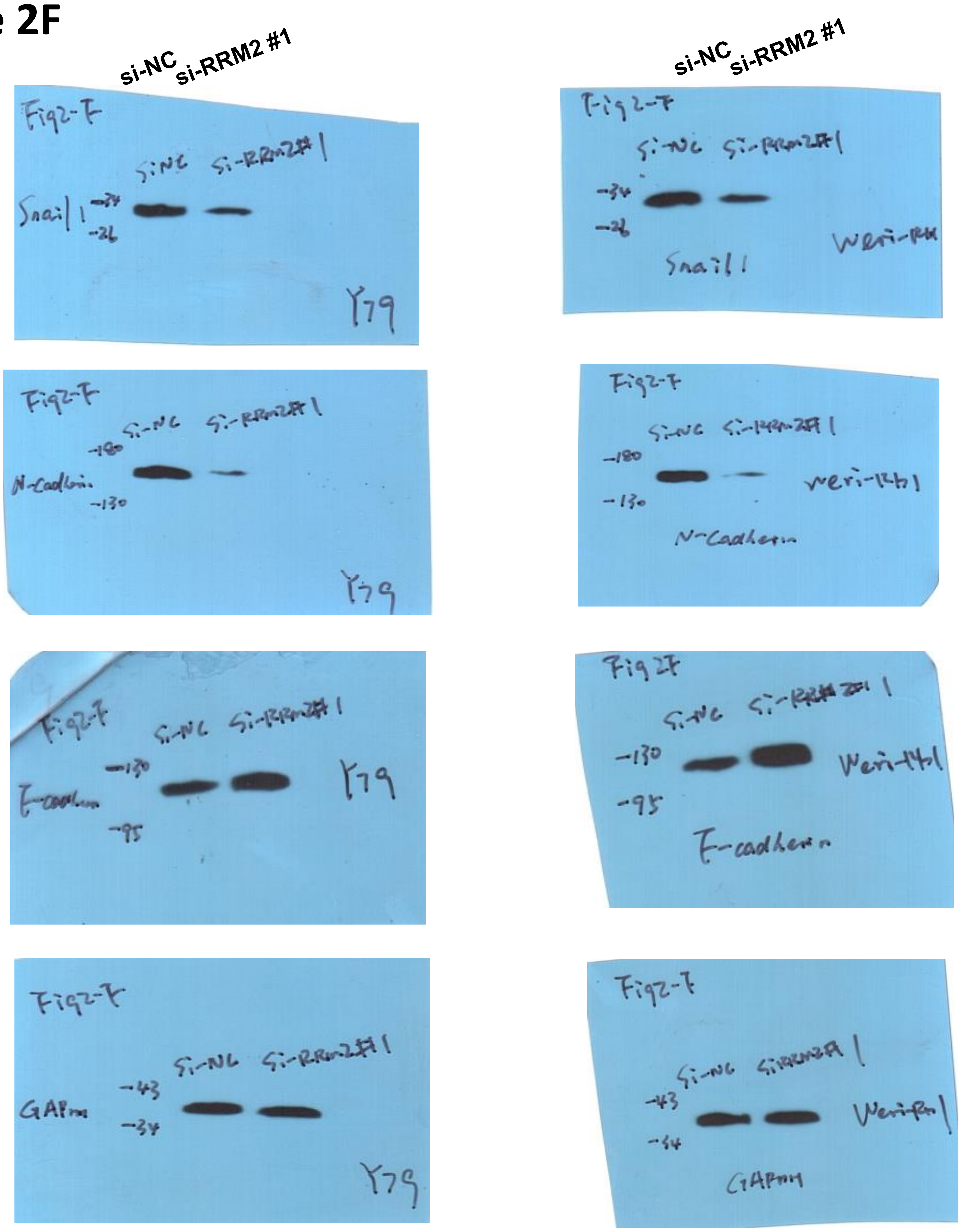

Figure 3A

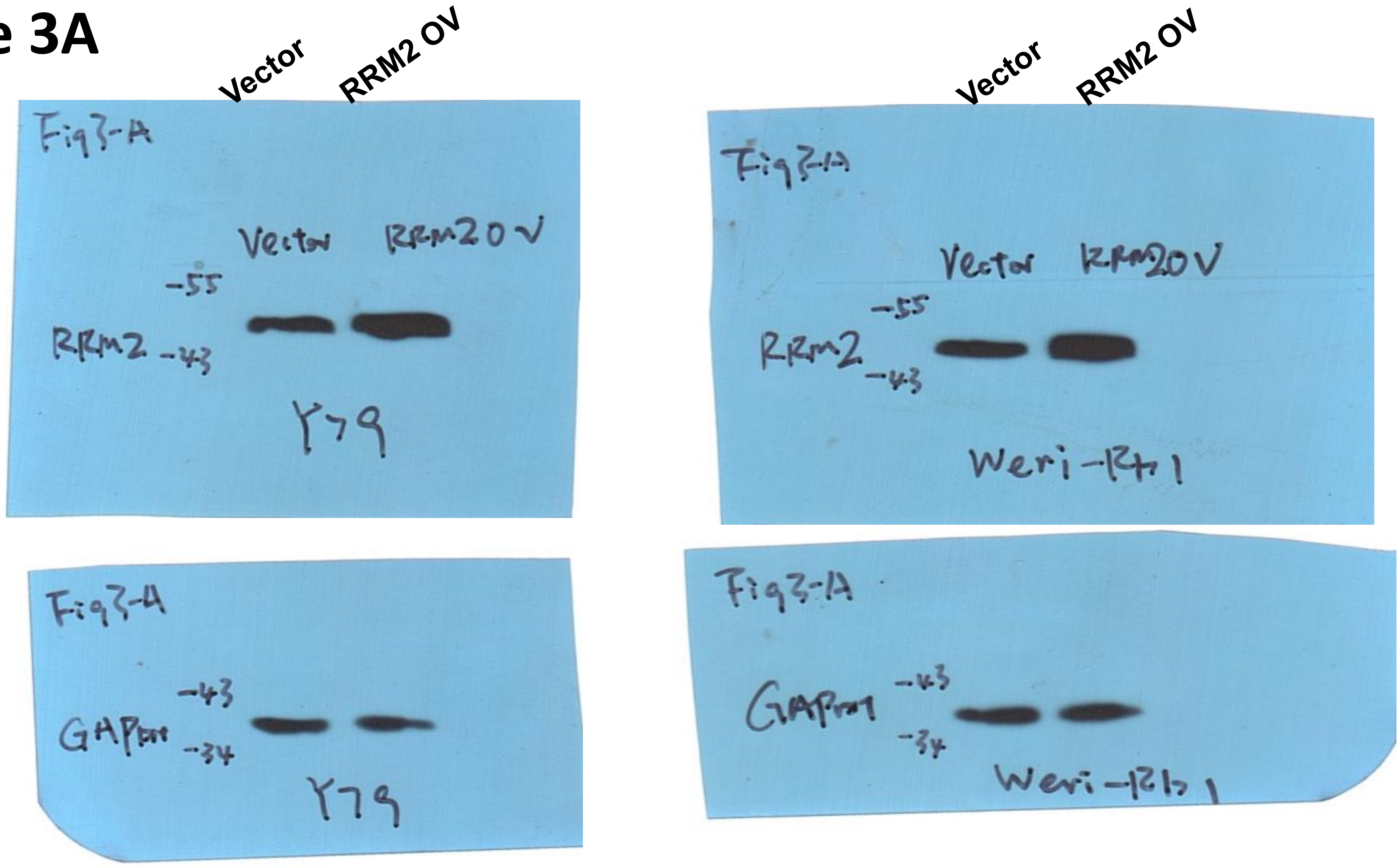

Figure 3F

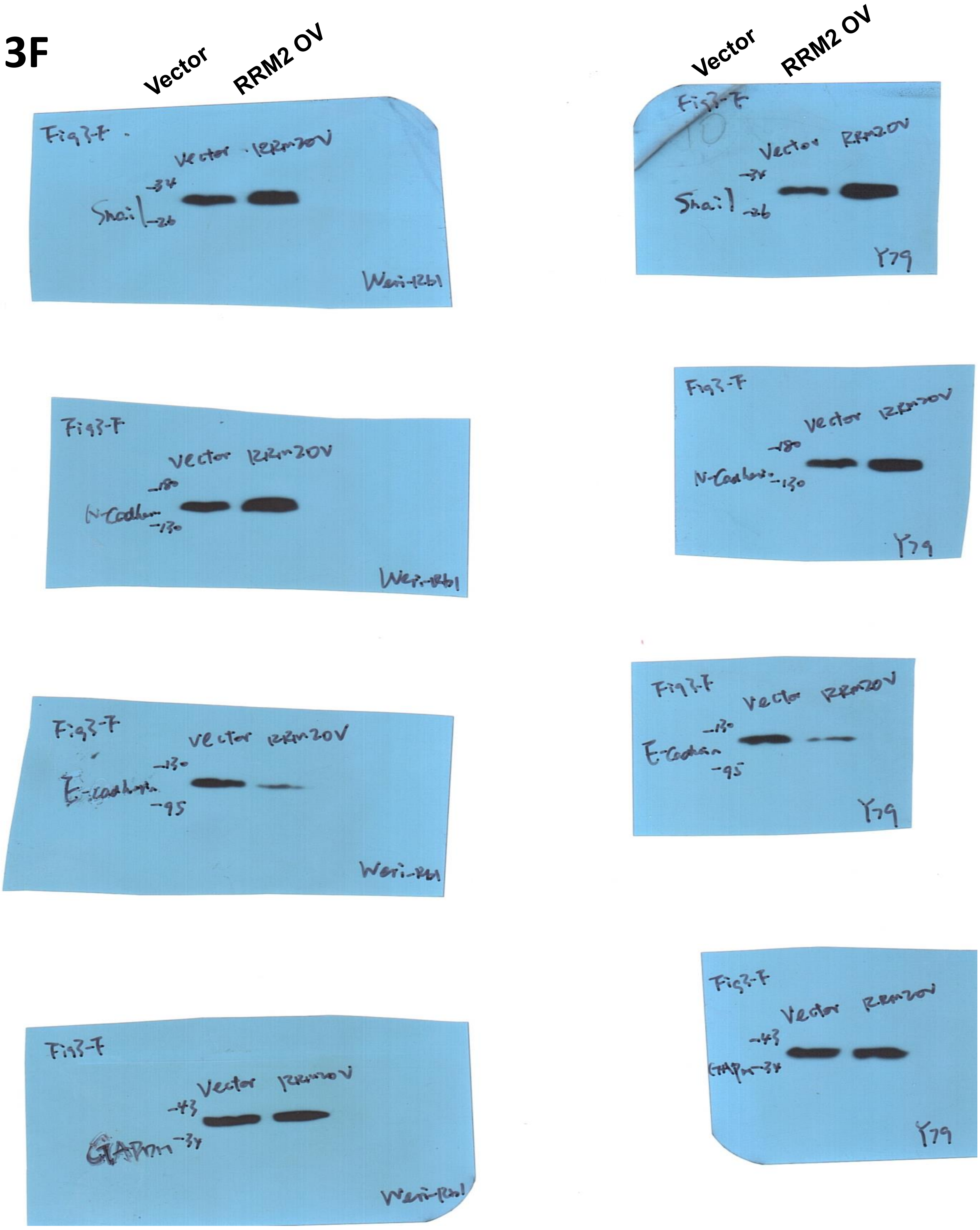

Figure 4A

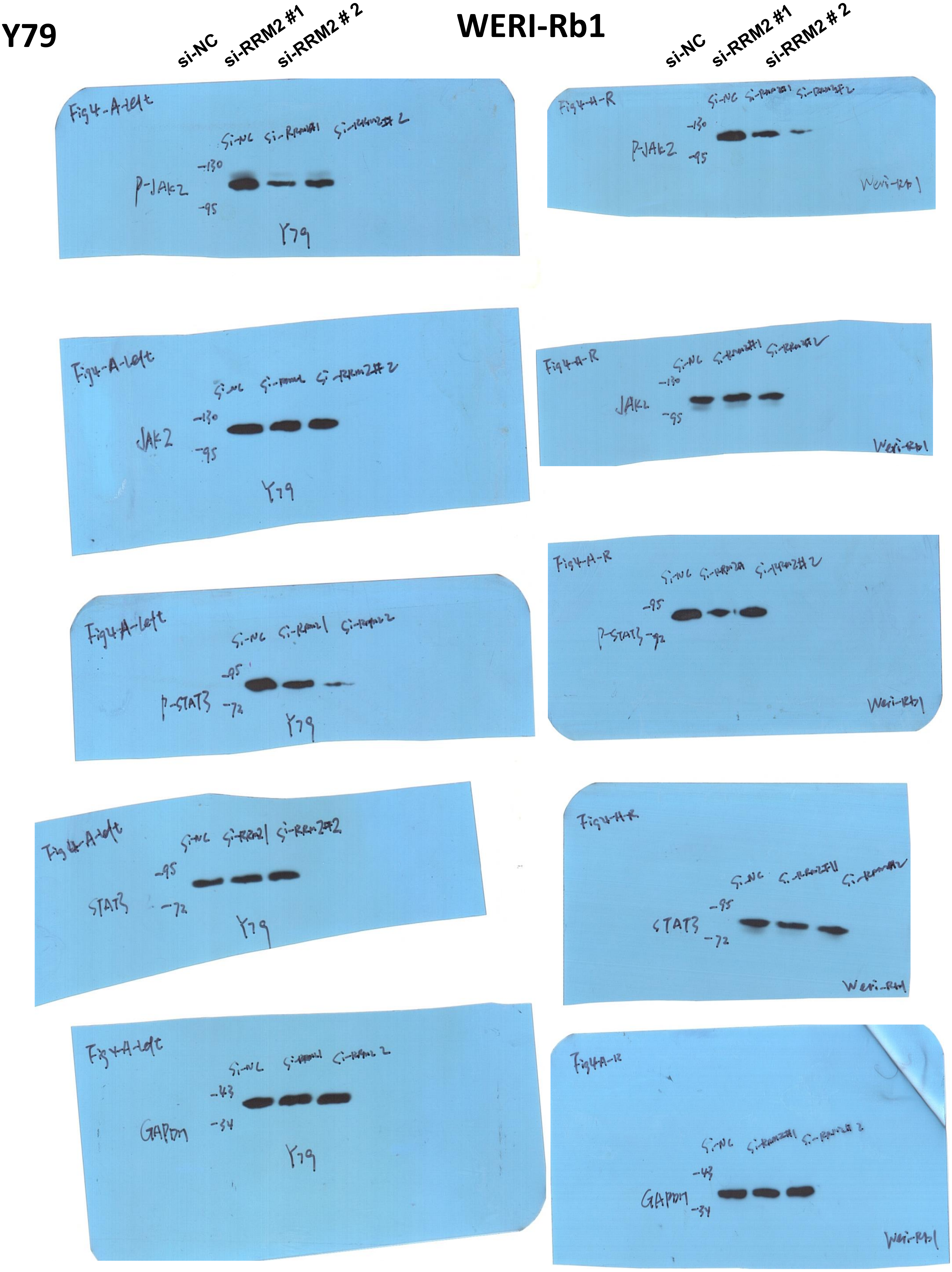

Y79
si-NC
si-RRM2 #1
si-RRM2 # 2
WERI-Rb1
si-NC
si-RRM2 #1
si-RRM2 # 2
p-JAK2
JAK2
p-STAT3
STAT3
GAPDH

Figure 4B

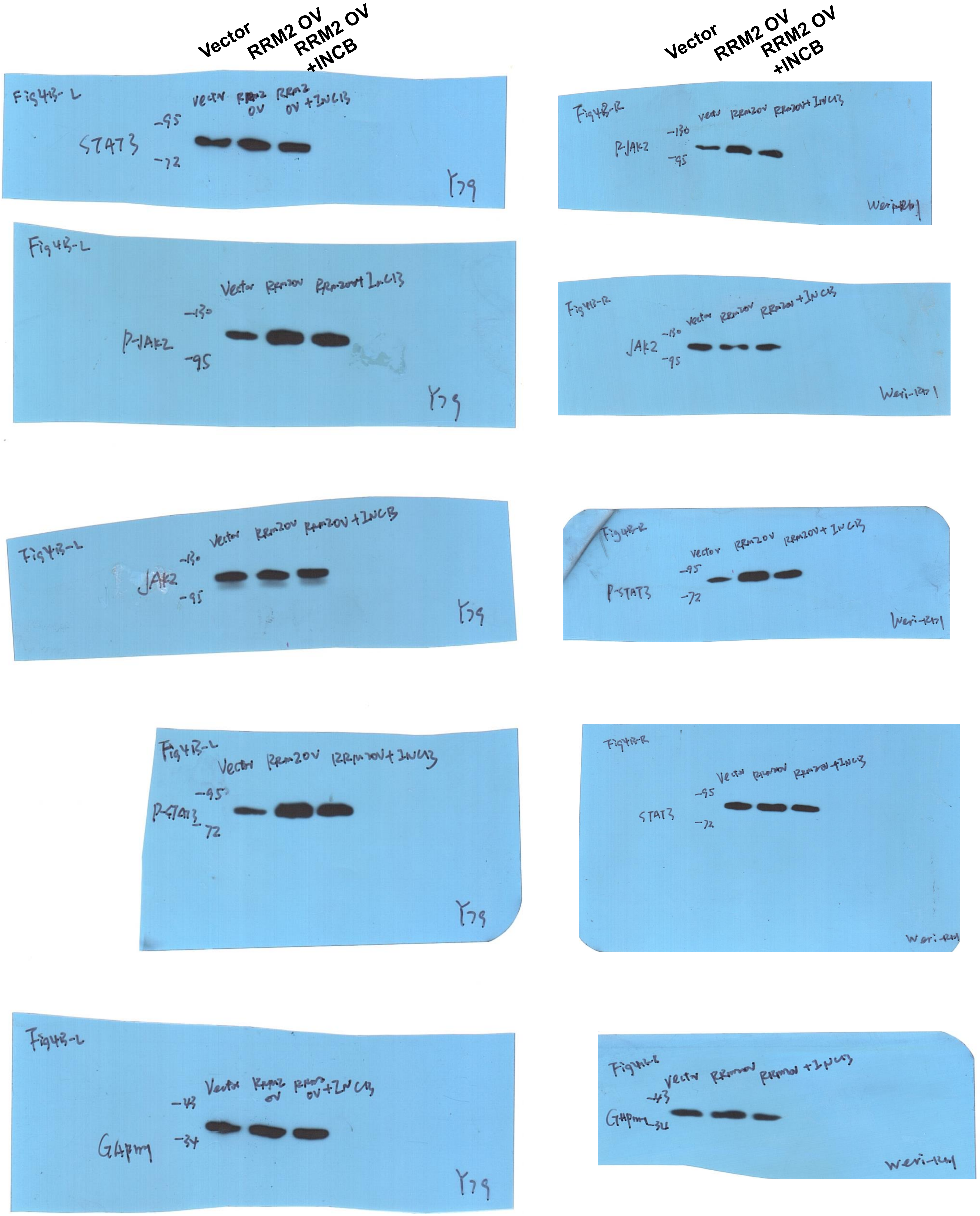

Figure 5C

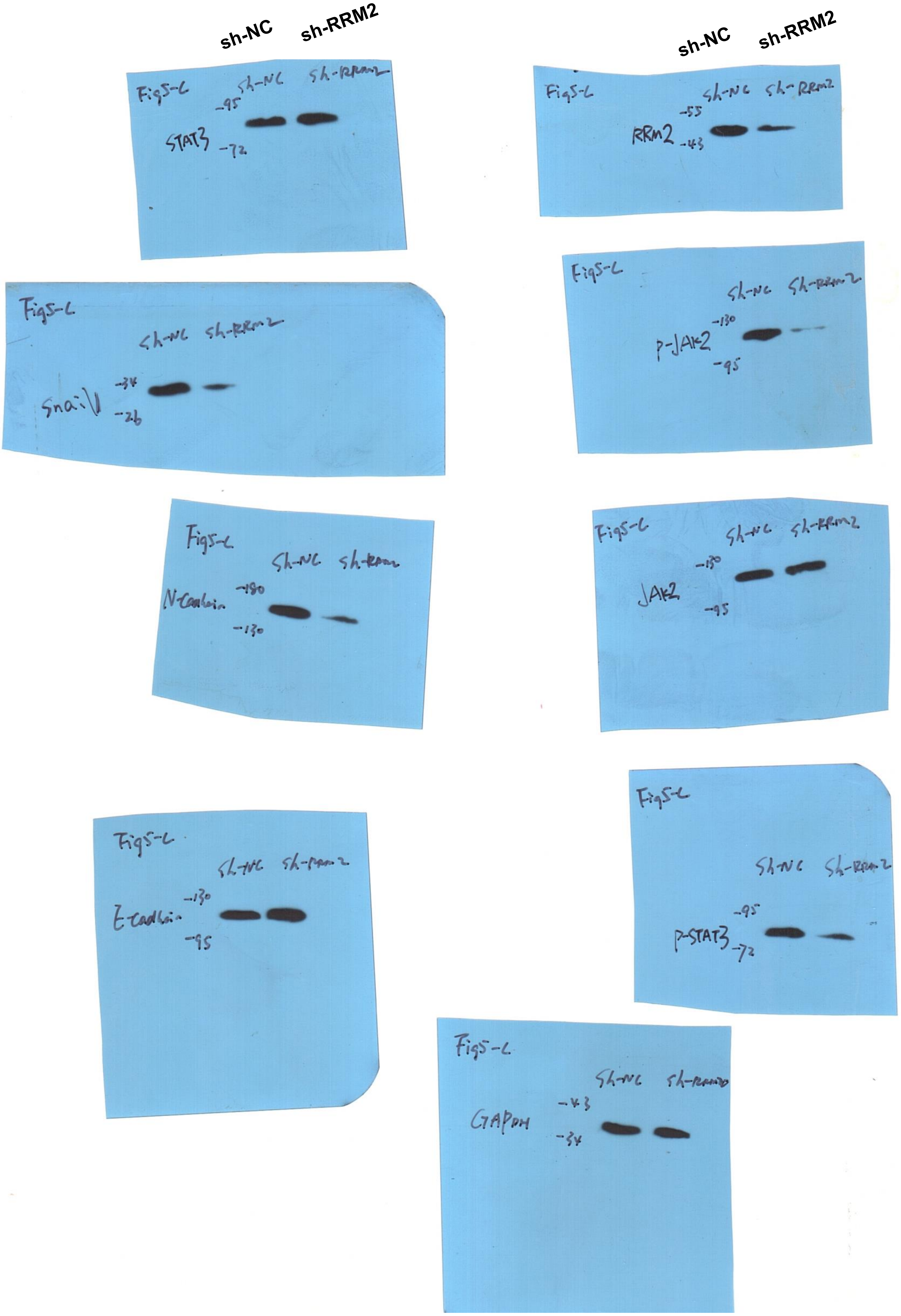
sh-NC
sh-RRM2
sh-NC
sh-RRM2
STAT3
RRM2
Snail1
p-JAK2
N-Cadherin
JAK2
E-Cadherin
p-STAT3
GAPDH
